# A new formulation of cannabidiol in cream shows therapeutic effects in a mouse model of experimental autoimmune encephalomyelitis

**DOI:** 10.1186/s40199-015-0131-8

**Published:** 2015-10-21

**Authors:** Sabrina Giacoppo, Maria Galuppo, Federica Pollastro, Gianpaolo Grassi, Placido Bramanti, Emanuela Mazzon

**Affiliations:** IRCCS Centro Neurolesi “Bonino-Pulejo”, Via Provinciale Palermo, contrada Casazza, 98124 Messina, Italy; Dipartimento di Scienze del Farmaco, Università del Piemonte Orientale, Largo Donegani 2, 28100 Novara, Italy; Consiglio per le Ricerca e la sperimentazione in Agricoltura – Centro di Ricerca per le Colture Industriali (CRA-CIN), Viale G. Amendola 82, 45100 Rovigo, Italy

**Keywords:** *Cannabis sativa* L, Multiple sclerosis, CBD-cream, Inflammation, Oxidative stress

## Abstract

**Background:**

The present study was designed to investigate the efficacy of a new formulation of alone, purified cannabidiol (CBD) (>98 %), the main non-psychotropic cannabinoid of Cannabis sativa, as a topical treatment in an experimental model of autoimmune encephalomyelitis (EAE), the most commonly used model for multiple sclerosis (MS). Particularly, we evaluated whether administration of a topical 1 % CBD-cream, given at the time of symptomatic disease onset, could affect the EAE progression and if this treatment could also recover paralysis of hind limbs, qualifying topical-CBD for the symptomatic treatment of MS.

**Methods:**

In order to have a preparation of 1 % of CBD-cream, pure CBD have been solubilized in propylene glycoland basic dense cream O/A. EAE was induced by immunization with myelin oligodendroglial glycoprotein peptide (MOG35–55) in C57BL/6 mice. After EAE onset, mice were allocated into several experimental groups (Naïve, EAE, EAE-1 % CBD-cream, EAE-vehicle cream, CTRL-1 % CBD-cream, CTRL-vehicle cream). Mice were observed daily for signs of EAE and weight loss. At the sacrifice of the animals, which occurred at the 28^th^ day from EAE-induction, spinal cord and spleen tissues were collected in order to perform histological evaluation, immunohistochemistry and western blotting analysis.

**Results:**

Achieved results surprisingly show that daily treatment with topical 1 % CBD-cream may exert neuroprotective effects against EAE, diminishing clinical disease score (mean of 5.0 in EAE mice vs 1.5 in EAE + CBD-cream), by recovering of paralysis of hind limbs and by ameliorating histological score typical of disease (lymphocytic infiltration and demyelination) in spinal cord tissues. Also, 1 % CBD-cream is able to counteract the EAE-induced damage reducing release of CD4 and CD8α T cells (spleen tissue localization was quantified about 10,69 % and 35,96 % of positive staining respectively in EAE mice) and expression of the main pro-inflammatory cytokines as well as several other direct or indirect markers of inflammation (p-selectin, IL-10, GFAP, Foxp3, TGF-β, IFN-γ), oxidative injury (Nitrotyrosine, iNOS, PARP) and apoptosis (Cleaved caspase 3).

**Conclusion:**

All these data suggest an interesting new profile of CBD that could lead to its introduction in the clinical management of MS and its associated symptoms at least in association with current conventional therapy.

**Electronic supplementary material:**

The online version of this article (doi:10.1186/s40199-015-0131-8) contains supplementary material, which is available to authorized users.

## Background

Cannabidiol (CBD) is the major non psychotropic constituent naturally present in *Cannabis sativa* L. plant isolated across the 1930s and 1940s, but chemically identified only in the 1960s by Mechoulam et al. [1]. As well documented from *Cannabis sativa* L. it is also possible to extract over 100 different cannabinoids compounds considered as its most important bioactive constituents and mainly known for their psychoactive effects [2]. Among these the main studied is the Δ^9^-tetrahydrocannabinol (Δ^9^-THC). This class of compounds have their effect mainly by interacting with specific receptors: the cannabinoid receptor type 1 (CB1), found on neurons and glial cells in various parts of the brain, and the cannabinoid receptor type 2 (CB2), found mainly in the body’s immune system [3, 4]. On the contrary, CBD has a very low affinity for these receptors (100 fold less than Δ^9^-THC) and when it binds it produces little to no effect [5].

CBD is able to exert multiple pharmacological actions via no-CB1 and no-CB2 receptors involving intracellular pathways that play a key role in neuronal physiology [6, 7]. In particular, many actions of CBD seem to be mediated by binding transient receptor potential vanilloid type 1 (TRPV1) [8], G protein-coupled receptor 55 (GPR55) [6, 9] and 5-hydroxytryptamine receptor subtype 1A (5-HT1A) [10]. These additional and novel cannabinoid receptors (no-CB1 and no-CB2) have been identified in CB1 and CB2- knockout mice and are expressed in both central and peripheral nervous system [11, 12].

Moreover, CBD has proved to have several anti-inflammatory activities and regulates cell cycle and immune cells functions [13]. CBD is able to suppress the production of a wide range of pro-inflammatory cytokines, such as tumor necrosis factor (TNF)-α and interleukin-1 beta (IL-1β), chemokines, growth factors, as well as inhibition of immune cell proliferation, activation, maturation, migration and antigen presentation [14, 15]. CBD shows also a potent action in inhibiting oxidative and nitrosative stress, modulating the expression of inducible nitric oxide synthase (iNOS) and nitrotyrosine as well as reducing production of reactive oxygen species (ROS) [16].

Just about all these properties showed by CBD, have prompted researchers to test its effects in a number of conditions involving both inflammation and oxidative stress, like neurodegenerative diseases, demonstrating in cell cultures as well as in animal models evident neuroprotective effects [16, 17].

Among this kind of disorders, multiple sclerosis (MS) is one of those obviously induced and driven by an unusual response of the immune system cells (T and B-lymphocytes) against myelin sheats of neurons [18]. During MS myelin autoreactive peripheral T cells migrate into the CNS and initiate cytotoxic, degenerative processes that include demyelination, oligodendrocyte cell death and axonal degeneration [15]. These effects lead to main clinical symptoms and neurological deficits [19].

According to the National MS Society, spasticity it is a common symptom in people suffering from MS. When MS damages the nerves that control muscles, it can result in spasticity that impairs movement and causes pain and stiffness. It usually occurs in the legs and can draw them up toward the body with painful cramping or cause spasms in the lower back, until losing the sensitivity of the limbs.

To date, current treatments for MS only offer palliative relief without providing a cure, and many are also associated with adverse effects that limit their long-term utility [20].

To overcome these limits, the interest of researchers was focused on finding alternative cure that could be less invasive and that may use for the treatment of MS and its correlated symptoms.

Numerous studies have been performed to evaluate the role of cannabinoids on treatment of EAE-associated spasticity as well as on modulation of the neurodegenerative process [21–25]. In this context, CBD has been proven to decrease peripheral inflammation and neuroinflammation in EAE mice when systemically given at the time of symptomatic disease [25]. In addition CBD is able to affect disease progression and ameliorated clinical symptoms. Moreover, CBD-Glatiramer Acetate (GA) combination administered in nasal delivery system (NDS) resulted in a statistically significant decrease of clinical scores and inflammatory cytokine expression in EAE mice [26].

Moreover, according to the two most relevant double-blind, randomized, placebo-controlled trials, benefits from use of cannabinoids seen in animal studies have also been shown in the treatment of MS patients suffering spasticity, with a significant associated disability and quality of life impairment [27, 28].

To date, the only commercially available preparation containing cannabinoids is Sativex® (GW Pharma, Ltd, Salisbury, Wiltshire, UK), an oral spray containing a mixture of two extracts in approximately a 1:1 ratio standardized to contain 2.7 mg of Δ^9^-THC and 2.5 mg of CBD/ 0.1 mL in an aromatized water-ethanol solution. Sativex® is used to alleviate spasticity in adult MS patients who do not show appropriate response to other drugs during an initial trial period of therapy [29].

Compared to other routes of administration, its advantage is a faster plateau of plasma concentration. Also, it has been established that coadministration of CBD and Δ^9^-THC can reduce unwanted effects of Δ^9^-THC [30].

The aim of this work was to study for the first time the effects of a topical administration of alone, purified CBD, as a new treatment strategy for MS. In specific, we evaluated whether treatment with a topical 1 % CBD-cream given at the time of symptomatic disease onset, could ameliorate the progression of the disease, counteracting the overall cascade of events occurring after EAE induction in mice. In addition, we investigated whether 1 % CBD-cream treatment could enhance responsiveness to a mechanical stimulus and recover paralysis of the hind limbs, qualifying topical-CBD for the symptomatic treatment of MS.

By examining this profile of CBD, we strongly hope to provide new evidences about the efficacy of the new topical treatment and to contribute into delineating a clearer profile of the compound so that its use could be an alternative to oral and parenteral administration of drugs for treatment of autoimmune and neurodegenerative diseases, like MS.

## Methods

### Plant material

*Cannabis sativa* L, derived from greenhouse cultivation at CRA-CIN, Rovigo (Italy), where a voucher specimen is kept, was collected in November 2013. The isolation and manipulation of cannabinoids was done in accordance with their legal status (Authorization SP/106 23/05/2013 of the Ministry of Health, Rome, Italy).

### Extraction and Isolation of CBD

Pure CBD (>98 %) was isolated from an Italian variety of industrial hemp (Carmagnola) according to the method of the cannabinoid purification reported in Taglialatela-Scafati O. et al. [31] with some modifications in order to avoid any trace of Δ^9^-THC that could interfere in the trial or causes legal limitation.

Dried flowerheads of *Cannabis sativa* (500 g) were heated at 120 °C in a ventilated oven for 2.5 h to decarboxylate pre-cannabinoids. After cooling to room temperature, the plant material was extracted with acetone (210 L). Removal of the solvent left a gummy residue that was partitioned between 1:1 aqueous methanol (1 L) and petroleum ether (1 L). The defatted polar phase was concentrated and extracted with CH_2_Cl_2_. The organic phase was dried (Na_2_SO_4_) and evaporated to afford a black gum (10 g), which was purified by flash chromatography on RP-18 silica gel (Biotage equipment, 250 mL column, linear gradient, from methanol water 55:45 to 90:10). Overall, five fractions were collected. The more polar one was further fractionated by gravity column chromatography on silica gel, with use of acidified (0.5 % HOAc) petroleum ether/EtOAc mixtures. After four chromatographic steps, crude CBD (10 mg) was obtained from a fraction directly eluted. The crude fraction was further purified by HPLC (eluentn-hexane/EtOAc 7:3) to provide pure CBD (6, 7.0 mg, 14 ppm based on dried plant material).

Finally the purity of CBD of 98 % was estimated by HPLC analysis according to the method of American Herbal Pharmacopoeia as reported in Swift et al. [32].

### Cream preparation

Pure CBD have been solubilized in propylene glycoland basic dense cream O/A to have a concentration of 1 % of CBD. Each application for both hind limbs regards a surface of about 1 cm^2^.

### Animals

Male C57BL/6 mice (Harlan Milan, Italy) 12 weeks of age and weighing 20–25 g were housed in individually ventilated cages with food and water *ad libitum*. The room was maintained at a constant temperature and humidity on a 12 h/12 h light/dark cycle.

### Ethics statement

This study was carried out in strict accordance with the recommendations in the guide for the care and use of laboratory animals of the National Institutes of Health. The protocol was approved by the Ministry of Health “General Direction of animal health and veterinary drug” (Authorization 150/2014-B 28/03/2014). In particular, animal care was in compliance with Italian regulations on protection of animals used for experimental and other scientific purposes (D.M. 116/92) as well as with the EEC regulations (O.J. of E.C.L 358/1 12/18/1986). Also, it was minimized number of animals used for this experiment and their suffering.

### Induction of Experimental Autoimmune Encephalomyelitis (EAE)

After anesthesia, induced with an anesthetic cocktail composed of tiletamine plus xylazine (10 ml/kg, ip), EAE was actively induced using Myelin Oligodendrocyte Glycoprotein peptide (MOG)_35–55_ (MEVGWYRSPFSRVVHLYRNGK; % peak area by HPLC ≥ 95, AnaSpec, EGT Corporate Headquarters, Fremont, CA, USA), according to Paschalidis et al. [33]. Mice were immunized subcutaneously with 300 μl/flank of the emulsion consisting of 300 μg of (MOG)_35–55_ in phosphate-buffered saline (PBS) mixed with an equal volume of Complete Freund’s Adjuvant (CFA) containing 300 μg heat-killed *M. Tubercolosis* H37Ra (Difco Laboratories Sparks, MD,USA). Immediately after (MOG)_35–55_ injection, the animals received an ip injection of 100 μl of *B. Pertussis* toxin (Sigma-Aldrich, Milan, Italy) (500 ng/100 μl, i.p), repeated 48 h later. The disease follows a course of progressive degeneration, with visible signs of pathology consisting of flaccidity of the tail and loss of motion of the hind legs.

### Experimental design

Mice were randomly allocated into the following groups (*N* = 40 total animals):

1. **Naive group** (*N* = 5): mice did not receive (MOG)_35–55_ or other treatment;
2. **EAE group** (*N* = 10): mice subjected to EAE that did not receive pharmacological treatment;
3. **EAE + 1** % **CBD-cream treatment group** (*N* = 10): EAE mice were subjected to one topical treatment of lower limbs with the 1 % CBD-cream every 24 h. In specific, before the beginning of treatment all animals were subjected to shaving of both hind limbs in outer thigh (area of 1 cm^2^) to facilitate the absorption of cream. Indeed, at each treatment cream was spread in this area until fully absorbed, also to prevent that animals could eat or lick cream. Only after the animals were placed in their cages. The treatment was started after the onset of disease signs and then daily protracted until the sacrifice;
4. **EAE + vehicle cream (no plus CBD) group** (*N* = 5): mice subjected to the same condition of the above group, but treated every 24 h with one topical application of the basic cream without CBD until the sacrifice;
5. **CTRL group + 1 % CBD-cream** (*N* = 5): mice subjected to shaving of both hind limbs in outer thigh (area of 1 cm^2^) and every 24 h subjected to one topical application with the 1 % CBD-cream that was spread until fully absorbed;
6. **CTRL group + vehicle cream (no plus CBD)** (*N* = 5): mice subjected to the same condition of the above group, but every 24 h treated with one topical application of the basic cream without CBD.

Of note, the last two groups were provided to verify if any beneficial effect was ascribed to the method of administration of the cream, such as spreading or whether treatment with 1 % CBD-cream or vehicle cream) could cause some allergic reaction in mice at cutaneous either at systemic level.

At the end of the experiment, which occurred at the 28^th^ day from EAE-induction, animals were euthanized with ip of Tanax (5 ml/kg body weight). Also, spinal cord tissues and spleen were sampled and processed in order to evaluate parameters of disease.

**Fig. 1:**
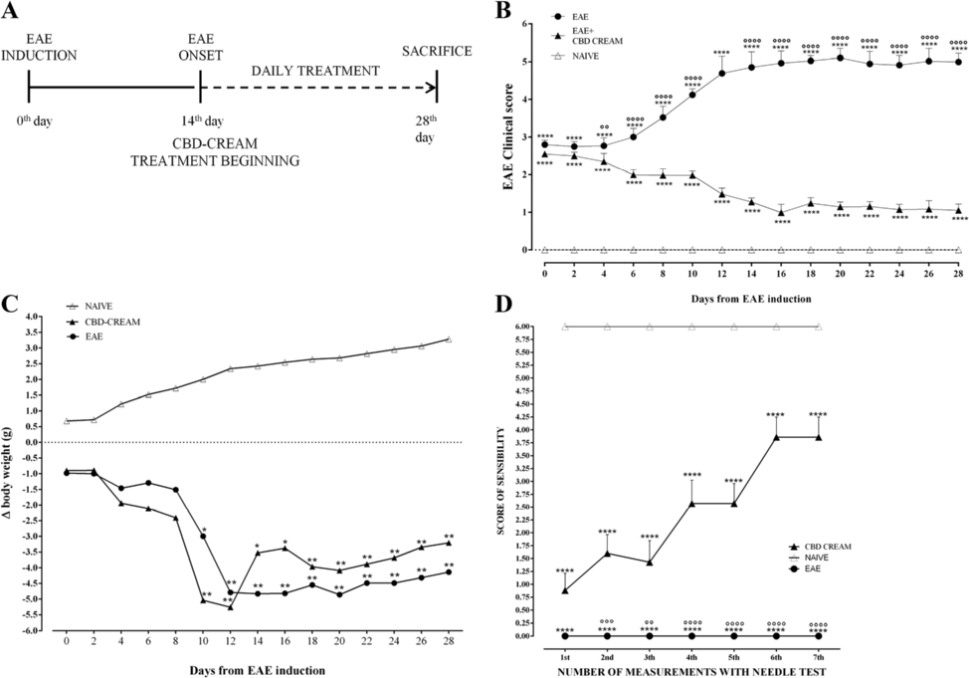
Panel **a** shows timeline of experimental design. EAE was induced on the 0^th^ day. The disease onset occurred on the 14^th^ day simultaneously daily treatment with CBD-cream was started and protracted until the day of sacrifice, which occurred at the 28^th^ day. Mice were immunized with MOG_35–55_ and monitored for clinical disease score of EAE (**b**) and body weight variations (**x**). Data have been expressed as mean ± SEM of all measurements of each experimental group. A p value < 0.05 was considered statistically significant. *p < 0.03 *vs* NAIVE, **p < 0.0011 *vs* NAÏVE (**b**). ****p < 0.0001 *vs* NAIVE, °°*p* < 0.0016, °°°°*p* < 0.0001 *vs* CBD-cream (**c**). Panel **d** displays score of sensibility measured by needle test ****p < 0.0001 *vs* NAÏVE, °°*p* < 0.0011, °°°*p* < 0.004, °°°°*p* < 0.0001 *vs* CBD-cream

Schematically, a plan of the experiment is shown in Fig. 1a.

### Clinical disease score and body weight evaluation

14 days after EAE induction, mice show the first signs of MS disease, characterized by loss of tail tonus, hind limb paralysis and body weight loss. Clinical score was evaluated according to a standardized scoring system [34] as follows: 0 = no signs; 1 = partial flaccid tail; 2 = complete flaccid tail; 3 = hind limb hypotonia; 4 = partial hind limb paralysis; 5 = complete hind limb paralysis; 6 = moribund or dead animal. Animals with a score ≥ 5 were sacrificed to avoid animal suffering.

The first measurement of clinical disease score and body weight were taken on the day of EAE- induction (day zero), and all the subsequent measurements were recorded every 48 h until sacrifice. Also, the daily variation of these two parameters of disease has been expressed compared to day of EAE induction (day zero). The value day has been expressed as mean ± SEM of all animals for each experimental group.

### Needle test

The test was aimed to assess mice’s responsiveness to a mechanical stimulus. It starts with the filament of 0.02 g, applying force to the left paw three times for a total period of 30 s (about 2 s to the stimulus) and to evaluate the response of the mouse after each application. The same treatment is repeated on the right paw. Response to two on three stimuli is regarded as a positive reaction. Specifically, a positive response is a paw withdrawal from the stimulus. The maximum score for both paws has a value of 6. In specific, 7 tests were performed every 48 h in two weeks from the first administration of 1 % CBD-cream. Animals were subjected to these measurements every 48 h in order to avoid additional stress that could affect the results. The values are expressed as mean ± SEM of each group.

### Blood sampling

At the time of sacrifice, following anesthesia blood samples were collected via cardiac injection in EDTA K2/gel tubes (BD Vacutainer® BD Diagnostic, Milan Italy) and centrifuged following at least 30 min from the collection at 10,000 g speed for 5 min. The achieved plasma was collected, aliquoted and stored at −20 °C to be used for detection of CBD.

### Pharmacokinetic analysis

Pharmacokinetc analysis of CBD plasma concentration was performed by liquid chromatography followed by mass spectrometry detection according to a reported method [38] with some modifications.

### May Grunwald Giemsa staining

At 28 days following EAE-induction, spinal cords were fully sampled from cervical to lumbar area and were fixed in 10 % (w/v) PBS-buffered formaldehyde. Spinal cord samples were first paraffin-embedded and cut into 7 μm-thick sections and then were deparaffinized with xylene, rehydrated, stained with May Grunwald Giemsa and studied using light microscopy (Leica ICC50HD microscope).

May Grunwald Giemsa staining was performed according to the manufacturer’s protocol (Bio-Optica, Milan, Italy) for the differentiation of cells present in lymphohemopoietic tissues. The staining solutions contain methylene blue (a basic dye), related azures (also basic dyes) and eosin (an acid dye). The first involves a blue/purple staining, the second involves a pink/red staining. The staining provides the nuclei of white blood cells and the granules of basophil granulocytes in blue, while red blood cells and eosinophil granules in red.

### Luxol Fast Blue (LFB)

To show myelin and phospholipids in histological sections, LFB staining was performed according to the manufacturer’s protocol (Bio-Optica, Milan, Italy). LFB affinity for central nervous system is usually ascribed to the bonds it forms with phospholipidic structures such as lecithin and sphingomyelin. The staining provides: myelin in turquoise blue, neurons and glial nuclei in pink/violet and Nissl substance in pale pink.

### Immunohistochemical evaluation

After deparaffinization with xylene, sections of spinal cord and spleen samples were hydrated. Detection of FOXP3, CD4, CD8α, GFAP, p-selectin, IL-1β, iNOS, nitrotyrosine and PARP-1 was carried out after boiling in citrate buffer 0.01 M pH 6 for 4 min. Endogenous peroxidase was quenched with 0.3 % (v/v) hydrogen peroxide in 60 % (v/v) methanol for 30 min. Nonspecific adsorption was minimized by incubating the section in 2 % (v/v) normal goat serum in PBS for 20 min.

Sections were incubated overnight with:

- anti-FOXP3 monoclonal antibody (1:100 in PBS v/v; Santa Cruz Biotechnology, Inc);
- anti-CD4 polyclonal antibody (1:100 in PBS v/v; Santa Cruz Biotechnology, Inc);
- anti-CD8α polyclonal antibody (1:100 in PBS v/v; Santa Cruz Biotechnology, Inc);
- anti-GFAP monoclonal antibody (1:50 in PBS v/v; Cell Signaling Technology);
- anti-p-selectin polyclonal antibody (1:100 in PBS v/v; Santa Cruz Biotechnology, Inc);
- anti-IL-1β polyclonal antibody (1:100 in PBS v/v; Santa Cruz Biotechnology, Inc);
- anti iNOS polyclonal antibody (1:100 in PBS v/v; Santa Cruz Biotechnology, Inc);
- anti-nitrotyrosine polyclonal antibody (1:100 in PBS v/v; Millipore);
- anti-PARP-1 polyclonal antibody (1:100 in PBS v/v; Santa Cruz Biotechnology, Inc).

Endogenous biotin or avidin binding sites were blocked by sequential incubation for 15 min with biotin and avidin (DBA, Milan, Italy), respectively. Sections were washed with PBS and incubated with secondary antibody. Specific labelling was detected with a biotin-conjugated goat anti-rabbit IgG and avidin–biotin peroxidase complex (Vectastain ABC kit, VECTOR). The immunostaining was developed with peroxidase substrate kit DAB (Vector Laboratories, Inc.) (brown color) and counterstaining with hematoxylin (blue background).

To verify the binding specificity, some sections were also incubated with only the primary antibody (no secondary) or with only the secondary antibody (no primary). In these cases no positive staining was found in the sections, indicating that the immunoreaction was positive in all the experiments carried out.

All sections were obtained using light microscopy (LEICA DM 2000 combined with LEICA ICC50 HD camera). Leica Application Suite V4.2.0 software was used as image computer program to acquire immunohistochemical pictures.

### Western blot analysis

All the extraction procedures were performed on ice using ice-cold reagents. In brief, spinal cord tissues were suspended in extraction buffer containing 0.32 M sucrose, 10 mM Tris–HCl, pH 7.4, 1 mM EGTA, 2 mM EDTA, 5 mM NaN_3_, 10 mM 2-mercaptoethanol, 50 mM NaF, protease inhibitor tablets (Roche Applied Science, Monza, Italy), and they were homogenized at the highest setting for 2 min. The homogenates were chilled on ice for 15 min and then centrifuged at 1000 g for 10 min at 4 °C, and the supernatant (cytosol + membrane extract from spinal cord tissue) was collected to evaluate content of cytoplasmic proteins.

The pellets were suspended in the supplied complete lysis buffer containing 1 % Triton X-100, 150 mM NaCl, 10 mM Tris–HCl, pH 7.4, 1 mM EGTA, 1 mM EDTA protease inhibitors (Roche), and then were centrifuged for 30 min at 15.000 g at 4 °C. Then, supernatant containing nuclear extract was collected to evaluate the content of nuclear proteins. Supernatants were stored at −80 °C until use. Protein concentration in homogenate was estimated by Bio-Rad Protein Assay (Bio-Rad, Segrate, Italy) using BSA as standard, and 20 μg of cytosol and nuclear extract from each sample were analyzed.

Proteins were separated on sodium dodecyl sulfate-polyacrylamide minigels and transferred onto PVDF membranes (Immobilon-P Transfer membrane, Millipore), blocked with PBS containing 5 % nonfat dried milk (PM) for 45 min at room temperature, and subsequently probed at 4 °C overnight with specific antibodies for TNF-α (1:500; Cell Signaling Technology), cleaved-caspase 3 (1:500; Cell Signaling Technology), GFAP (1:1000; Cell Signaling Technology), IL-6 (1:500; Abcam), IL-10 (1:250; Santa Cruz Biotechnology Inc), TGF-β (1:500; Abcam) and IFN-γ (1:250; Santa Cruz Biotechnology Inc) in 1x PBS, 5 % (w/v) non fat dried milk, 0.1 % Tween-20 (PMT). HRP-conjugated goat anti-mouse IgG, HRP-conjugated goat anti-rabbit IgG or HRP-conjugated chicken anti-rat were incubated as secondary antibody (1:2000; Santa Cruz Biotechnology Inc) for 1 h at room temperature.

To ascertain that blots were loaded with equal amounts of protein lysates, they were also incubated with antibody for GAPDH HRP Conjugated (1:1000; Cell Signaling Technology) and beta-actin (1:1000; Santa Cruz Biotechnology, Inc). The relative expression of protein bands, was visualized using an enhanced chemiluminescence system (Luminata Western HRP Substrates, Millipore) and protein bands were acquired and quantified with ChemiDoc™ MP System (Bio-Rad) and a computer program (ImageJ software) respectively.

Blots are representative of three separate and reproducible experiments. The statistical analysis was carried out on three repeated blots performed on separate experiments.

### Statistical evaluation

GraphPad Prism version 6.0 program (GraphPad Software, La Jolla, CA) was used for statistical analysis of the data. The results were statistically analyzed using one-way ANOVA followed by a Bonferroni *post hoc* test for multiple comparisons. A *p* value less than or equal to 0.05 was considered significant. Results are expressed as the mean ± SEM of n experiments.

## Results

### Pharmacokinetic parameters of CBD after topical cream application

The summary of the pharmacokinetic parameters is showed in Table 1. The results of the *in vivo* experiment showed that the steady-state plasma concentration (C_ss_) of CBD were 6.1 ± 1.9 ng/mL, which were attained at 14.9 ± 12.0 h (T_lag_). Also, the maximum plasma concentration (C_max_) was 8.3 ± 2.1 ng/mL and the temperature maximum (T_max_) was 38.2 ± 18.9. Statistical analysis was performed by one-way analysis of variance followed by Bonferroni *post hoc* analysis. A *p* value less than or equal to 0.05 was considered significant. Data represent mean ± SD.

**Table 1:** Pharmacokinetic parameters of CBD after topical treatment with 1 % CBD-cream

Pharmacokinetic parameter	CBD
C_max_ (ng/mL)	8.3 ± 2.1
T_max_ (hours)	38.2 ± 18.9
C_ss_ (ng/mL)	6.1 ± 1.9
T_lag_ (hours)	15.5 ± 12.0

### Clinical score and body weight

Clinical disease score (Fig. 1b) as well as body weight measurement (Fig. 1c) evaluation was assessed as parameters of disease. In both cases, CBD-treated EAE-affected mice show a trend of recovery over time compared to untreated EAE mice, in particular following the disease onset and until sacrifice. As displayed, mice belonging to the EAE group show a grading of disease with a clinical score as mean of 5.0 ± 0.329, while mice treated topically with 1 % CBD-cream revealed a lower grade of disability with a clinical score as mean of 1.5 ± 0.214. (Fig. 1b). In addition, in mice pharmacologically treated the recovery of the clinical score matches with an increase of body weight. Moreover, as expected, after EAE induction, a significant body weight loss was observed in EAE mice. Mice belonging to naive group have a normal increase in body weight as well as absence of motor deficit.

These data confirm both the disability in mice affected by EAE and the belief that chronic inflammation and autoimmune conditions in animals are associated with substantial feeding alterations.

Of note during the entire treatment period were not detected in mice treated with 1 % CBD-cream or vehicle cream (no plus CBD) allergic reactions in mice at cutaneous either at systemic level. Also, in order to confirm this, mice were subjected to every 48 h to little samples of blood from the tail to make a blood smear glass slide and calculate subsequent leukocyte formula, demonstrating any alteration in percentage of leukocytes.

### CBD enhances responsiveness to a mechanical stimulus

Mice subjected to EAE show many clinical and pathological features of human MS, like paralysis of the hind limbs. In order to evaluate whether topical treatment with CBD can improve limbs sensitivity to a mechanical stimulus, we measured by needle test mechanical allodynia that may arise after peripheral nerve injury (Fig. 1d). To this purpose, a stimulus was applied on the paw plantar surface, evaluating the paw retraction response treshold.

In EAE mice treated topically with CBD a significantly increased response to mechanical stimulus was evident already from the first measurements. Moreover, EAE mice showed no response to mechanical stimulus applied to the paw plantar, while naive animals always responded by retracting the paw (Fig. 1d).

### CBD improves histopathology of EAE

**Fig. 2:**
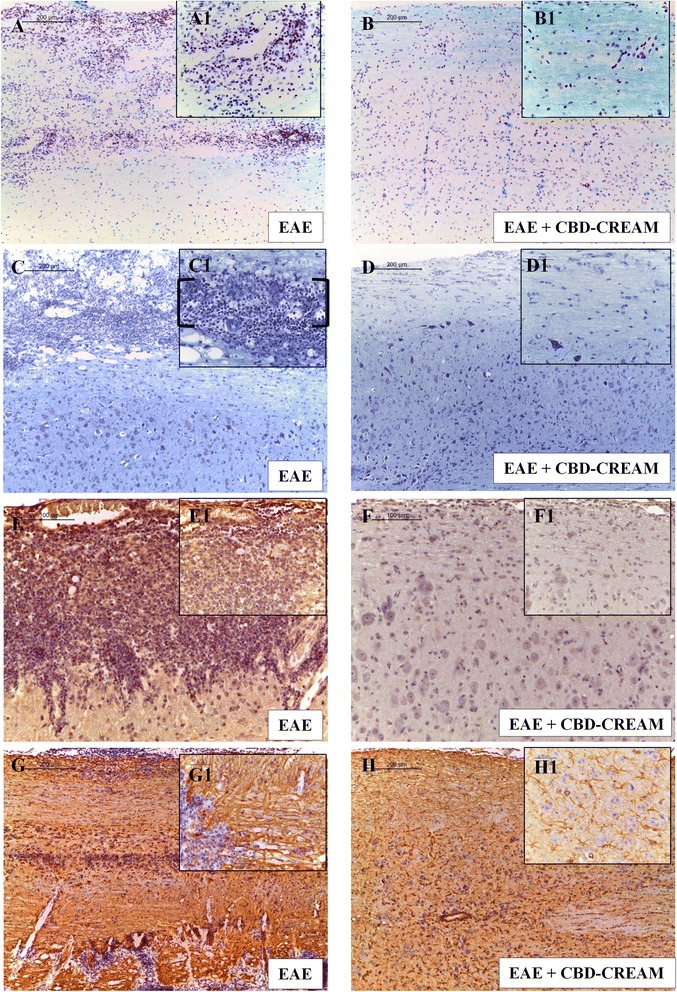
LFB staining compared EAE group (**a**:10x, A1 magnification:40x) to EAE+ 1 % CBD-cream (**b**:10x, B1 magnification:40x). May-Grunwald Giemsa staining for EAE mice (**c**:10x, C1 magnification:40x) compared to mice treated with 1 % CBD-cream (**d**:10x, D1 magnification:40x). Immunohistochemical evaluation for Foxp3 in EAE (**e**:20x, E1 magnification:40x) and in EAE + 1 % CBD-cream mice (**f**:20x, F1 magnification:40x). Immunohistochemical evaluation for GFAP in EAE (**g**:10x, G1 magnification:40x) and in EAE + 1 % CBD-cream mice (**h**:10x, H1 magnification:40x)

Since EAE is a demyelinating disease, CBD was evaluated for the protective effect on myelin sheath integrity by LFB staining at 28 days after EAE induction. Compared to naive animals and CTRL + CBD-cream (Additional files 1A and B, EAE mice exhibited markedly reduced myelin and axonal structures in the spinal cord (Fig. 2a). Also, treatment with 1 % CBD-cream, reduced demyelination and axonal loss in EAE mice with a high LFB positive staining (Fig. 2b).

The May-Grunwald Giemsa staining method was also used to evaluate inflammatory cells infiltration. The observed results clearly show considerable infiltration of lymphocytes cells in white matter of spinal cord samples taken from EAE mice (Fig. 2c, as shownn by square brackets) compared with naive mice and CTRL + CBD-cream (Additional files 1C and D). Remarkably, treatment with CBD-cream led to a complete resolution of inflammatory cells infiltration (Fig. 2d).

### CBD modulates production of Treg cells and CD4 and CD8α Tcells

To assess whether CBD was able to modulate the production of Treg cells, we evaluated expression of the transcription factor Foxp3 by immunohistochemical analysis. Spinal cord sections from naive mice and CTRL + CBD-cream (Additional files 1E and F) did not show positive staining for Foxp3, which conversely, was positive in EAE mice (Fig. 2e). Sections obtained from 1 % CBD-cream treated group showed negative degree for Foxp3 (Fig. 2f).

**Fig. 3:**
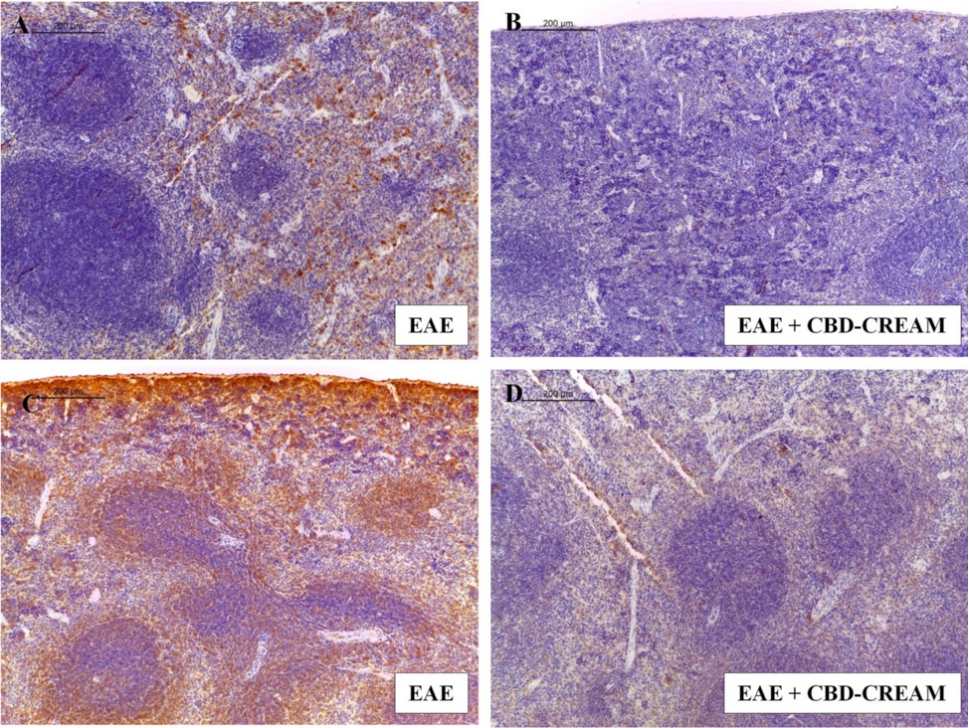
Immunohistochemical analysis for CD4 in spleen tissues from EAE mice (**a**:10x, A1 magnification:40x) and mice treated with 1 % CBD-cream (**b**:10x, B1 magnification:40x). Immunohistochemical image for CD8α localization of EAE mice (**c**:10x, C1 magnification:40x) compared to CBD topical treated mice (**d**:10x, D1 magnification:40x) in spleen tissues

In addition, to these observations, immunohistochemical analysis carried out in spleen sections, showed a positive staining for CD4 as well as for CD8α in EAE mice (Fig. 3a and b). Conversely, a negative staining for CD4 and CD8α was observed in EAE mice administered with 1 % CBD-cream (Fig. 3c and d) as well as in naïve mice and CTRL + CBD-cream (Additional files 2E, F, G and H). Also, overall quantitative analysis of immunohistochemical images showed that tissue localization for CD8α was estimated about 35.96 % of positive staining in EAE mice, 3.63 % in EAE + 1 % CBD-cream mice and 0 in naïve group. Also, for CD4 tissue localization was estimated about 10.69 % of positive staining in EAE mice, 2.53 % in EAE + 1 % CBD-cream mice and 1.02 % in naïve ones. These images are representative of at least three experiments. Values shown are the mean of three different fields observed.

### Effects CBD on GFAP expression

In order to investigate whether CBD can modulate astrocytic activation, we evaluated GFAP expression by immunohistochemical analysis. GFAP is considered a marker protein for astrogliosis, and a marked positive staining for GFAP was evident in the sections from EAE mice (Fig. 2g), compared to naive group and CTRL + CBD-cream (Additional files 1G and H). GFAP positive staining was significantly reduced in animals treated topically with CBD (Fig. 2h). Also, the same results were corroborated by western blot analysis on spinal cord tissues (Fig. 5e).

### CBD modulates p-selectin expression

**Fig. 4:**
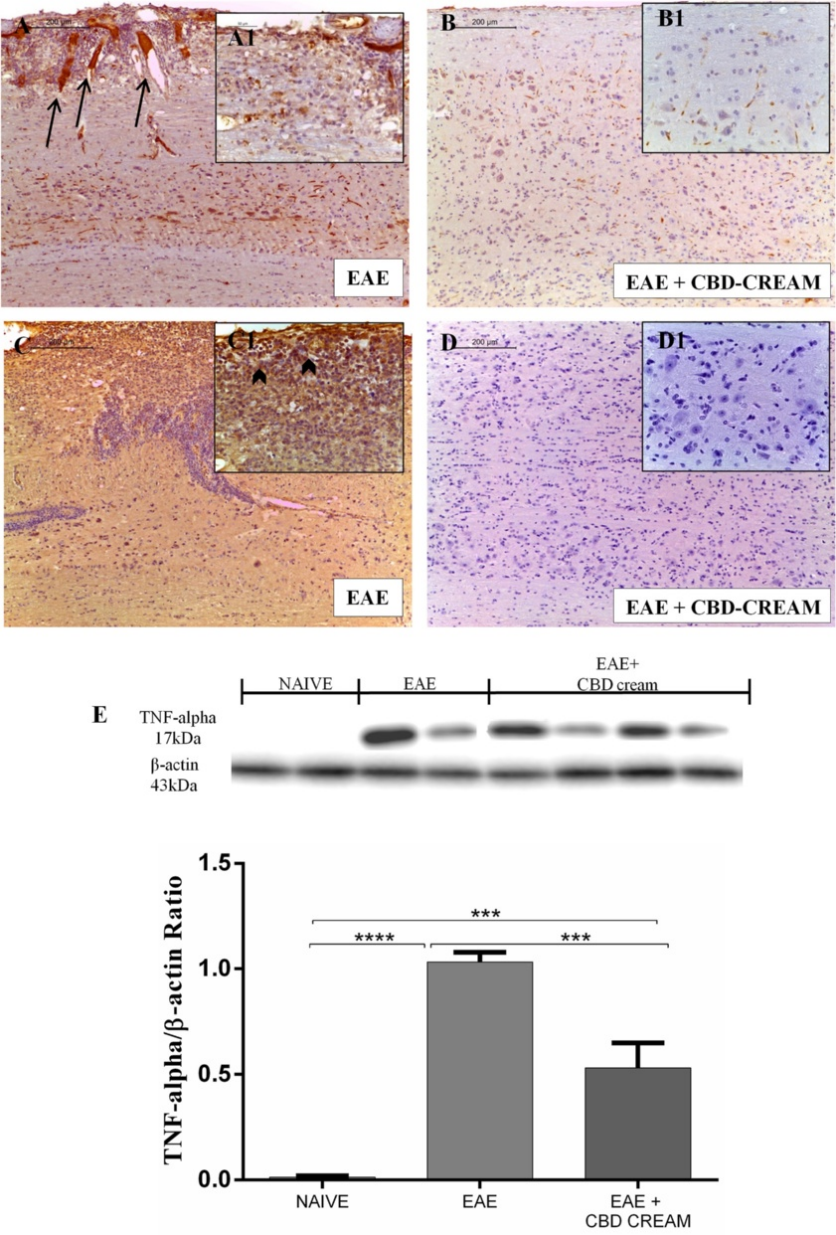
Comparision of p-selectin immunohistochemical localization between EAE mice (**a**:10x, A1 magnification:40x) and EAE treated mice with 1 % CBD-cream (**b**:10x, B1 magnification:40x). Immunohistochemical analysis for IL-1β in spinal cord tissues from EAE mice (**c**:10x, C1 magnification:40x) and mice treated with 1 % CBD-cream (**d**:10x, D1 magnification:40x). Western blot analysis for TNF-α was showed in **e**. β-actin was used as internal control. ****p < 0.0001 *vs* EAE, ***p < 0.0004, ***p < 0.0010 *vs* EAE + 1 % CBD-cream

No positive staining for p-selectin was observed in longitudinal sections of spinal cord from naive mice and CTRL + CBD-cream (Additional files 1A and B), whereas an intense positive staining in the vascular endothelium of EAE mice (Fig. 4a as shown by arrows) was observed. Conversely, negative staining for p-selectin was observed in spinal cord tissues from mice treated with 1 % CBD-cream (Fig. 4b).

### CBD regulates inflammatory pathway

In order to investigate whether treatment with CBD can modulate the inflammatory processes triggered by EAE induction through regulating secretion of pro-inflammatory cytokines, the expression levels of IL-1β, IL-6, TNF-α, TGF-β and INF-γ in spinal cord samples were quantified by immunohistochemical and western blot analysis, respectively.

A positive staining for IL-1β was observed in spinal cord sections of EAE mice group (Fig. 4c arrowheads indicate positivity for inflammatory cells, including lymphocytes and neutrophils) when compared to naive mice (Additional files 1C and D), while no positive staining for cytokine expression was obtained in mice treated with CBD-cream (Fig. 4d). Additionally, by western blot analysis on homogenates of spinal cord tissues, a considerable increase in TNF-α release was established in EAE mice compared to naive animals. Conversely, levels of TNF-α were attenuated by 1 % CBD-cream administration. (Fig. 4e).

**Fig. 5:**
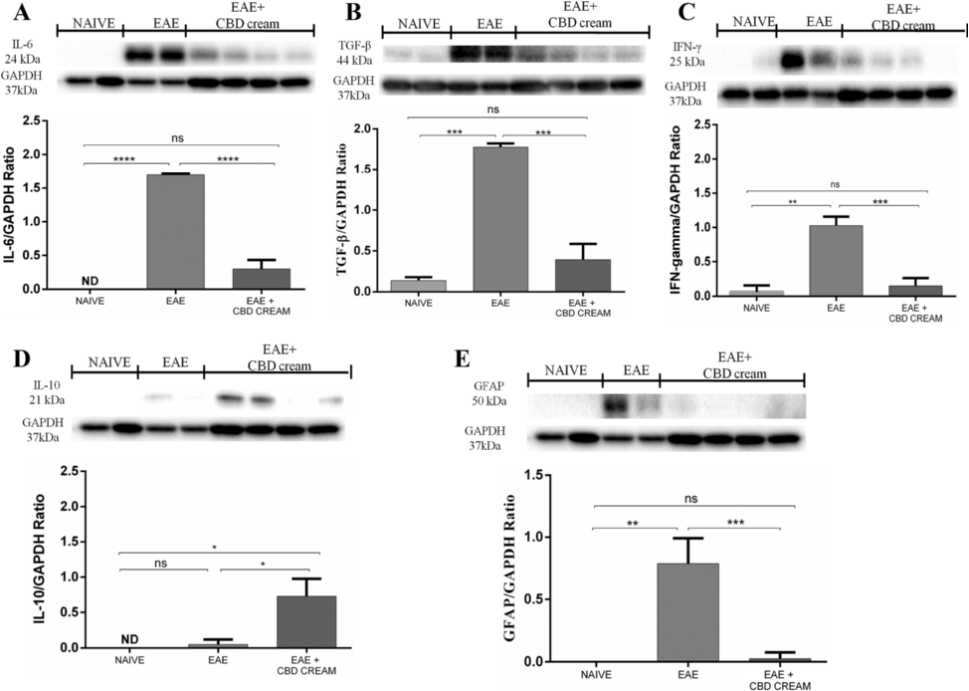
Western blot analysis for IL-6 was showed in **a**. ****p < 0.0001 *vs* EAE, ****p < 0.0001 *vs* EAE + 1 % CBD-cream. Western blot analysis for TGF-β (**b**). ***p < 0.0004 *vs* EAE, ***p < 0.0004 *vs* EAE + 1 % CBD-cream. In **c** was displayed western blot analysis for IFN-γ. **p < 0.0011 *vs* EAE, ***p < 0.0008 *vs* EAE + 1 % CBD-cream. Western blot analysis for IL-10 (**d**). *p < 0.0326, *p < 0.0415 *vs* EAE + 1 % CBD-cream. In **e** was displayed western blot analysis for GFAP **p < 0.0016 *vs* EAE, ***p < 0.0009 *vs* EAE + 1 % CBD-cream. ND not detectable

By Western blot, we found also appreciably increased expression of IL-6 in the spinal cord tissues from EAE mice On the contrary, topical treatment with 1 % CBD-cream diminished its levels (Fig. 5a).

Western blot analysis for TGF-β revealed that this signaling pathway is strongly activated following EAE-induction while 1 % CBD-cream treatment reduces the expression levels of this marker (Fig. 5b). Similarly, it was found an increased expression of INF-γ in EAE mice, decreased by 1 % CBD-cream treatment (Fig. 5c).

Also, by western blot analysis we investigated the role of IL-10 as antinflammatory cytokine, showing a basal level of IL-10 expression in samples obtained from EAE mice, whereas treatment of mice with 1 % CBD-cream significantly increased its expression. It was not observed an expression of IL-10 in naïve animals. (Fig. 5d).

### CBD modulates production of nitrotyrosine, iNOS and PARP

**Fig. 6:**
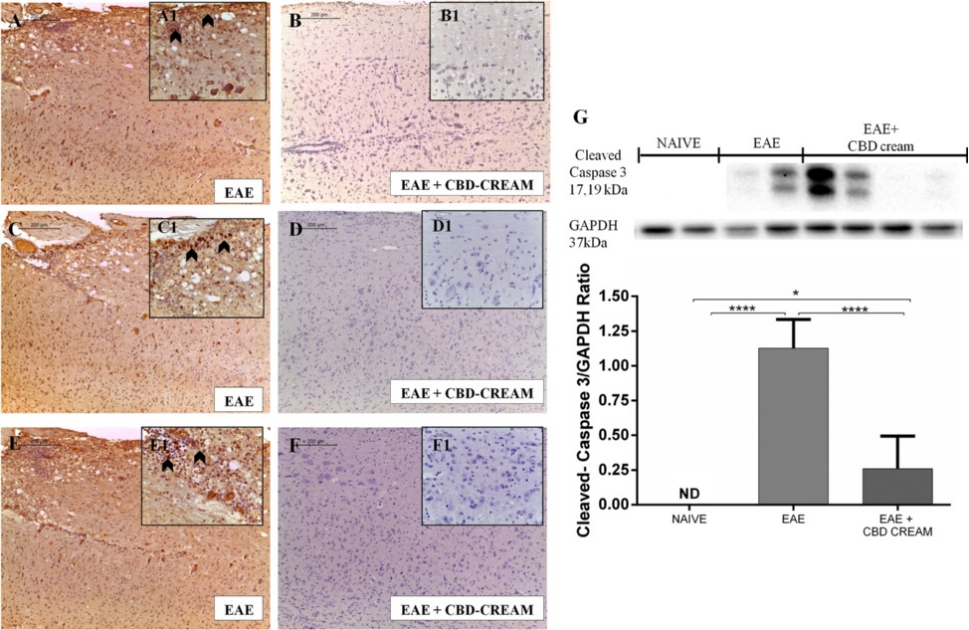
Immunohistochemical image for nitrotyrosine localization of EAE mice (**a**:10x, A1 magnification:40x) compared to CBD topical treated mice (**b**:10x, B1 magnification:40x). Immunohistochemical evaluation for iNOS in EAE (**c**:10x, C1 magnification:40x) and in EAE + 1 % CBD-cream mice (**d**:10x, D1 magnification:40x). Immunohistochemical analysis for PARP in spinal cord tissues from EAE mice (**e**) and mice treated with 1 % CBD-cream (**f**). Panel **g** shows western blot analysis for Cleaved-caspase 3. GAPDH was used as internal control. ****p < 0.0001 *vs* EAE, *p < 0.0244, ****p < 0.0001 *vs*. EAE + CBD-cream. ND not detectable

CBD could also counteract the nitrosative stress resulting from the EAE-induction. Spinal cord sections obtained from EAE untreated mice exhibited positive staining for nitrotyrosine (Fig. 6a as shown by arrowheads), iNOS (Fig. 6c arrowheads shown positivity for inflammatory cells) and PARP (Fig. 6e as shown by arrowheads) .Sections obtained from mice treated with 1 % CBD-cream showed negative staining for nitrotyrosine (Fig. 6b), iNOS (Fig. 6d) as well as PARP staining (Fig. 6f).

### CBD reduces Cleaved-caspase 3 expression induced by EAE

By Western blot, we evaluated the activation of the caspase pathway, particularly, cleaved-caspase 3, which leads to programmed cell death by cleavage of cellular substrates. Cleaved-caspase 3 levels were appreciably increased in the spinal cord tissues from EAE mice. On the contrary, topical treatment with 1 % CBD-cream prevented EAE-induced cleaved-caspase 3 production (Fig. 6g).

## Discussion

*Cannabis sativa* represents a great source of bioactive compounds whose potential for medicinal use is currently at the center of an intense research activity, especially for the management of neurodegenerative disorders, and MS in particular.

In this regard it is noteworthy to consider the recent introduction of Sativex®, a cannabinoid oromucosal spray containing a 1:1 ratio of THC and cannabidiol CBD, for the management of symptomatic treatment of chronic pain and spasticity in MS patients [29]. Despite this combination has been approved and in the current state introduced in several countries under this formulation, over the years the point of view of the scientific community regarding THC and CBD is changed. Although emerging evidence regarding putative therapeutic activities of cannabinoids, to date it remains to be overcome limits about unavoidable psychotropic effects, exhibited by many of them [30].

Several experimental studies have shown that CBD possesses many properties, often wrongly attributed by collective imagination just to THC alone and wide experimental evidences demonstrated that isolating non-psychotropic compounds by THC component provide beneficial effects for therapeutic use, mostly for CNS disorders [35, 36].

To date, although CBD pharmacodynamic remains still unclear, its pharmacokinetics appears better defined. It is known that when orally given, due to a marked first-pass effect, CBD bioavailability ranges between values of 13 and 19 % [37], hence the oral route is not ideal for the therapeutic delivery of CBD, but for this reason the intravenous administration is preferable. Even further intranasal administration could be more effective as results in the rapid attainment of the drug blood level, but it is more suitable for acute or breakthrough pain [38].

As CBD has low aqueous solubility and undergoes first-pass metabolism, alternative delivery routes would be necessary to achieve successful therapeutic effects.

An easy method of CBD administration would be through the topical route, thus maintaining a costant therapeutic drug level and reducing increased side effects because of the decreased peak plasma levels. It has been demonstrated, although only a few published studies, that cannabinoids are good candidates for topical delivery in the treatment of chronic conditions [39, 40].

For this reason, the present work was designed to define a new topical formulation of CBD, whose intrinsic potential as a molecule with a therapeutic effect has not yet completely understood.

Recent studies have already described beneficial effects of intraperitoneal administration of CBD alone or in mixture with other compounds in EAE mouse model with antinflammatory and antioxidant properties [23–25]. According to Rahimi et al. [41] intraperitoneally treatment with CBD in mixture with palmitoylethanolamide (PEA) during EAE onset reduced the severity of the neurobehavioral deficits of EAE. This effect of CBD and PEA was accompanied by decreased inflammatory cytokines expression, demyelination, axonal damage and inflammatory cytokine expression [41].

Also, in Kozela et al. study [25] it was found that systemic treatment with alone CBD during disease onset ameliorated the severity of the clinical signs of EAE. This effect of CBD was accompanied by diminished axonal damage and inflammation as well as microglial activation and T-cell recruitment in the spinal cord of EAE mice. Also, this effect seems not to be mediated via the known cannabinoid CB1 and CB2 receptors [25].

Similarly, our achieved results have demonstrated that topical formulation of CBD significantly modulates many intracellular pathways associated to EAE/MS etiopathology, by improving clinical features correlated with severity of the pathology.

One of the first hallmarks of disease in the MOG-induced EAE model is a reduction of body weight and onset of symptoms such as tail tonus and hind limb paralysis. As expected, mice belonging to EAE group showed the highest score of disease (about 5 points in the grading scale of disease) against 1,5 of EAE topical treated mice. The therapeutic effect in terms of wellness following the CBD administration as topical treatment was a reduced body weight loss associated with an improvement in the disease score. Therefore, 1 % CBD-cream has proven effective into reduce the main disease parameters.

Moreover, as known, EAE is associated with a complex series of processes triggered by neuroinflammation and the infiltration of immune cells into the CNS, followed by a dysfunction of neural activity and neuronal death [42]. Here, we confirmed findings of above cited studies, demonstrating that 1 % CBD-cream treatment acts counteracting leukocyte infiltration and microglial activation and improving neuroinflammation status. Hystopathology of spinal cord samples supported this view showing a marked remyelination following 1 % CBD-cream treatment. To better understand this aspect, we believe usefull to focus the attention on the above cited leukocyte infiltration key-event of EAE model and critical step into the establishment of inflammatory response and demyelinization [ 43].

In this context, adhesion molecules, as p-selectin, play a significant role in the induction of leukocyte-endothelial cell adhesion and extravasation of immune cells during pathological process [44]. Therefore, according to a supposed action on this pathway mediated by 1 % CBD-cream treatment, it was not surprising to find a modulated p-selectin immunolocalization.

Although as known that MS is a disease mediated by an autoimmune attack directed against components of the myelin sheath, the mechanisms that lead to loss of function associated with these immunologically events remain poorly understood. The activation of T cells and macrophages that secrete freely diffusable factors has an important role in the pathogenesis of MS. These factors are the proinflammatory cytokines and reactive oxygen and reactive nitrogen species [45, 46]. As expected we found increased levels of proinflammatory mediators including IL-1β, TNF-α, IL-6, IFN-γ and TGF-β in spinal cord from EAE mice. Remarkably, 1 % CBD-cream treatment significantly attenuates the expression of all these inflammatory markers and also regulates immune tolerance by increasing the production of antinflammatory cytokines, like IL-10. This confirms that CBD was able to decrease TNF-α, IL-2 and IFN-γ release from activated splenocytes and macrophages as reported in other studues [47–49].

Of note, this cytokine profile suggests it as an important factor in immunopathogenesis of MS, because the main feature of MS pathophysiology is the neuroinflammatory reaction.

As reported in literature it’s probably that all these mediators are produced by Th17 cells, that play a key role in in autoimmune neuroinflammation and EAE development [50]. Also, it is generally considered that Th17-mediated inflammation is characterized by neutrophil recruitment into the CNS and neurons killing. In this context, mainly IFN-γ seems to have a pathological role in the development of this autoimmune disease [51]. Our results show that the MOG-induced EAE causes significant increases in IFN-γ expression. 1 % CBD-cream administration, significantly diminished the expression of this cytokine.

Moreover, several studies on MS evaluated immunosuppressive effect of cannabinoids demonstrated that they are able to exert their action modulating the induction of Tregulatory cells (Tregs) [13], normally involved in the maintenance of tolerance toward self-constituents, and limiting inflammatory responses against foreign antigens. Treg cell recruitment plays a key defensive role in suppression of Th1 effector cells, which are the main T cell subtype mediating disease pathogenesis.

To verify if treatment with CBD can modulate the production of Treg cells, we evaluated the expression of the transcription factor Foxp3, as an indirect marker of Treg. In accordance with a previous study [52], we confirmed a clear engagement of Treg cells during EAE, while treatment with CBD-cream is able to deplete Foxp3 positive cells. This leads to think that the immune system response is somehowe restrained by topic application of 1 % CBD-cream in EAE affected mice in correlation with a lower degree of autoimmune cells activity in these animals. Also, it is possible that 1 % CBD-cream stimulates Th0 cell to develop into a Treg phenotype.

This observation has special relevance if we consider that CD4 T cells expression is involved in cell-mediated immunity and in the pathogenesis of MS, with destruction of the axonal myelin sheath in several areas of CNS and spinal cord being mediated mainly by self-reactive CD4 T cells. As expected, we observed that both CD4 and also CD8α detections were apparent in untreated EAE mice, while topical treatment reveals the capability to counteract the release of cytotoxic T cells.

Corroborating these results, immunostaining for GFAP, a marker of astrocytic activation, involved in many processes of cellular function [53], was reduced in EAE mice treated with 1 % CBD-cream compared with untreated mice. This potentially can be correlated with the triggering of antioxidant mechanisms that, notoriously, interfere with GFAP upregulation occurring in astocytes.

Looking at this first report of achieved results we feel to state that CBD, acting as an antinflammatory agent, is capable to reduce the inflammatory signs of MS. Nevertheless, we have obtained deeper data characterizing the therapeutic properties of the 1 % CBD-cream preparation.

For this, we have to consider that CBD has a potent capability to attenuate oxidative and nitrosative stress in wide neurological disease models, although the underlying mechanism is still unclear.

As, it is known that several cellular mediators and proinflammatory cytokines can induce iNOS, enzyme present in actively demyelinating lesions, and that nitrates and nitrites are increased in the CSF and serum of patients with MS [54]. Therefore, several studies have suggested a role for NO and its oxidizing molecules (such as peroxynitrite) in the immunopathogenesis of MS as contributors of the inflammatory process [55] In accordance with these assumptions, we demonstrated that CBD reduces the expression of iNOS and nitrotyrosine in tissues from EAE treated mice, suggesting that this compound, at least in part, may be responsible for the reduction of cytokines production, that in turn counteract the rise of iNOS levels and the downstream cascade of events triggered by the inflammatory process, reducing thus oxidative stress.

The strong link between oxidative stress and apoptosis is well known, as is the concept that down-regulation of nitrosative stress and protection against apoptosis within the CNS represents an effective neuroprotective therapy for the MS treatment. In particular, PARP expression is correlated and in turn activated by cleaved-caspase 3. Positive staining for PARP, responsible of DNA breakdown in apoptosis, was found in EAE mice, while it was significantly reduced by topical treatment with CBD. In keeping with these observations, the expression of cleaved caspase 3, a major key regulator of apoptosis, was evaluated displaying an attenuated expression when CBD was topically administered.

All these results overall suggest and allow us to hypothesize that CBD has the capability to interfere with EAE-induced neuronal apoptotic death, attenuating, or even preventing the activation of cellular molecular mechanisms triggered by severe damage.

Moreover, in the current literature CBD has been shown to be a potent analgesic in animal models of hyperlgesia and mechanical allodynia, as possible candidate for the treatment of inflammatory pain and other common symptoms related to chronic pain [56]. These analgesic effects of CBD might be mediated by its modulation of TRPV1 receptor [57]. Furthermore, there are evidences that cannabinoids induce antinociception both via supraspinal mechanisms and the interaction with peripheral CB2 receptors and via activation of CB1 receptors at the spinal cord level [58]. However, CBD shows only marginal affinity for CB1 and CB2, and the potential involvement of the endocannabinoid system might be indirectly mediated by its effects on the enzymes of the endocannabinoid system.

We also confirmed that CBD has a beneficial effect on mechanical allodynia in EAE animals. Since mice subjected to EAE show paralysis of the hind limbs with a consequent loss of sensitivity, we evaluated the effects of a CBD topical treatment applied on the hind limbs of animals in recovering a response to a mechanical stimulus induced on the paw plantar surface. Amazingly, topical CBD significantly increased response to mechanical stimulus from the earliest measurements, recovering the responsiveness of the hind limbs.

We can also affirm that the protective effects of treatment are not due to the method of drug administration (shaving and/or spreading of the cream). Animals treated with cream that not containing the active ingredient did not show any improvement in recovering paralysis of the hind limbs. For us, this represent a data is of pivotal importance, and in the event that it will be clinically confirmed, this finding could open new opportunities for the treatment of MS.

## Conclusions

Summarizing, we have shown that the topical administration of CBD can protect against the cascade of events (inflammation, oxidative injury and neuronal cell death) associated to the induction of EAE. Of note, topical CBD application was able to recover the hind limb lost sensitivity. This observation provides a rationale for evaluating its clinical translation that might represent a new concept in the management of MS.

Finally, we suggest that CBD, devoid of psychoactive activity, could be potentially, safe and effective non invasive alternatives for alleviating neuroinflammation and neurodegeneration.

## Additional files

Additional file 1: Figure S1. LFB staining shows naive group (A:10x) and CTRL-CBD cream (B:10x). May-Grunwald Giemsa staining for naive mice (C:10x) and mice CTRL-CBD cream (D:10x). Immunohistochemical evaluation for Foxp3 in naive group (E:10x) and in CTRL-CBD cream (F:10x). Immunohistochemical evaluation for GFAP in naive mice (G:10x) and in mice CTRL-CBD cream (H:10x). (TIFF 18429 kb) Additional file 2: Figure S2. Immunohistochemical localization for p-selectin in naive mice (A:10x) and CTRL-CBD cream (B:10x). Immunohistochemical analysis for IL-1β in spinal cord tissues from naive mice (C:10x) and for CTRL-CBD cream (D:10x). Immunohistochemical analysis for CD4 in spleen tissues from naive mice (E:10x) and for CTRL-CBD cream (F:10x). Immunohistochemical image for CD8α localization of naive mice (G:10x) and for CTRL-CBD cream (H:10x) in spleen tissues. (TIFF 19661 kb)

## Abbreviations

CBD: Cannabidiol
EAE: Autoimmune encephalomyelitis
MS: Multiple sclerosis
MOG_35–55_: Myelin oligodendrocyte glycoprotein peptide
Δ^9^-THC: Δ^9^-tetrahydrocannabinol
CB1: Cannabinoid receptor type 1
CB2: Cannabinoid receptor type 2
TRPV1: Transient receptor potential vanilloid type 1
GPR55: G protein-coupled receptor 55
5-HT1A: 5-hydroxytryptamine receptor subtype 1A
TNF-α: Tumor necrosis factor-alpha
IL-1β: Interleukin-1beta
iNOS: Inducible nitric oxide synthase
ROS: Reactive oxygen species
GA: Glatiramer acetate
NDS: Nasal delivery system
CFA: Complete Freund’s adjuvant
LFB: Luxol Fast Blue
C_SS_: steady-state plasma concentration
Cmax: maximum plasma concentration
T_max_: temperature maximum
PEA: palmitoylethanolamide
Tregs: regulatory T cells
