# Supplementary figures and images for: A new formulation of cannabidiol in cream shows therapeutic effects in a mouse model of experimental autoimmune encephalomyelitis
Source: Daru. 2015 Oct 21;23:48. doi: 10.1186/s40199-015-0131-8 (PMC4618347; doi:10.1186/s40199-015-0131-8)

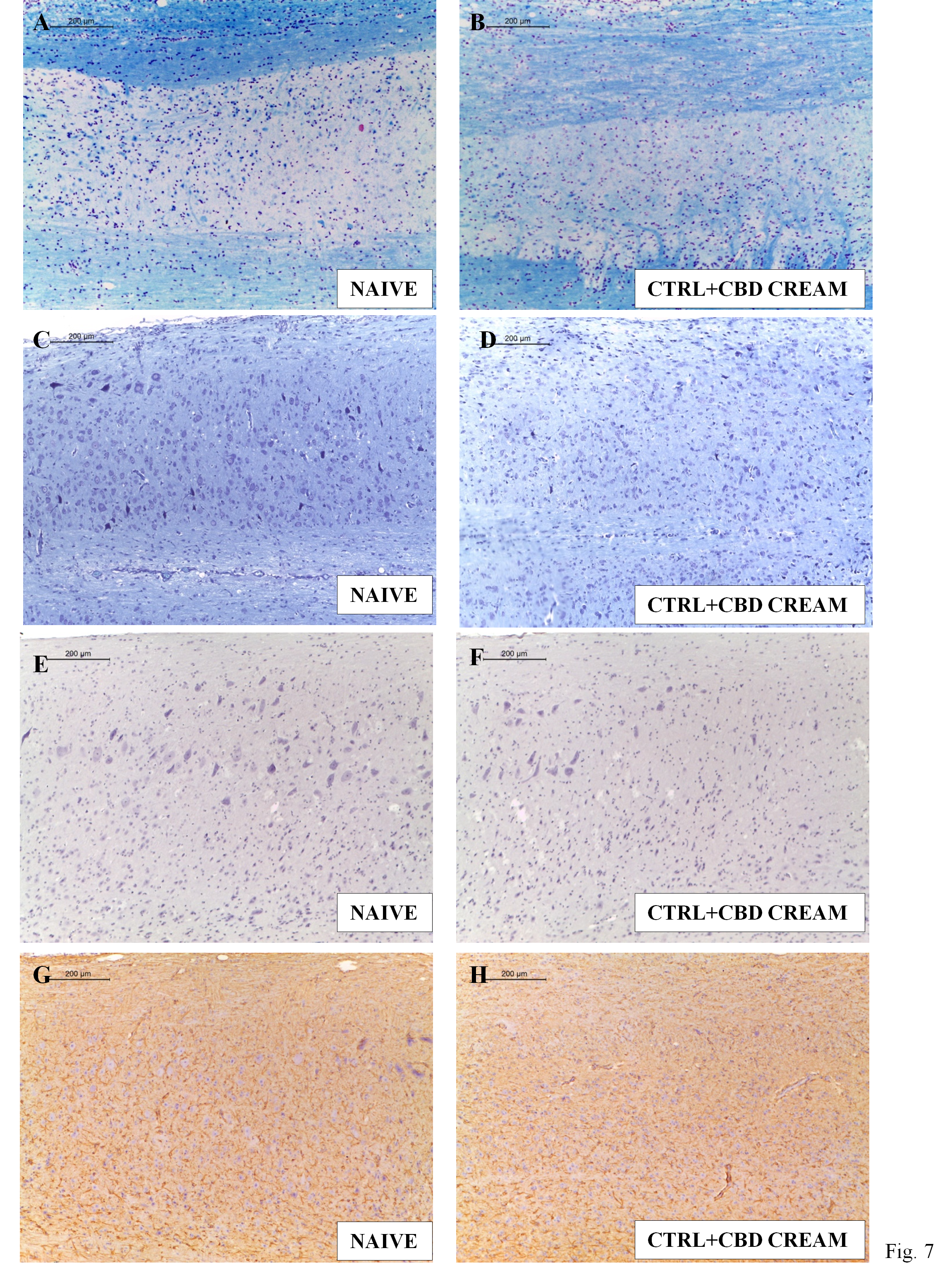

Supplement: Additional file 1: Figure S1. — LFB staining shows naive group (A:10x) and CTRL-CBD cream (B:10x). May-Grunwald Giemsa staining for naive mice (C:10x) and mice CTRL-CBD cream (D:10x). Immunohistochemical evaluation for Foxp3 in naive group (E:10x) and in CTRL-CBD cream (F:10x). Immunohistochemical evaluation for GFAP in naive mice (G:10x) and in mice CTRL-CBD cream (H:10x). (TIFF 18429 kb) [file 40199_2015_131_MOESM1_ESM.tif]

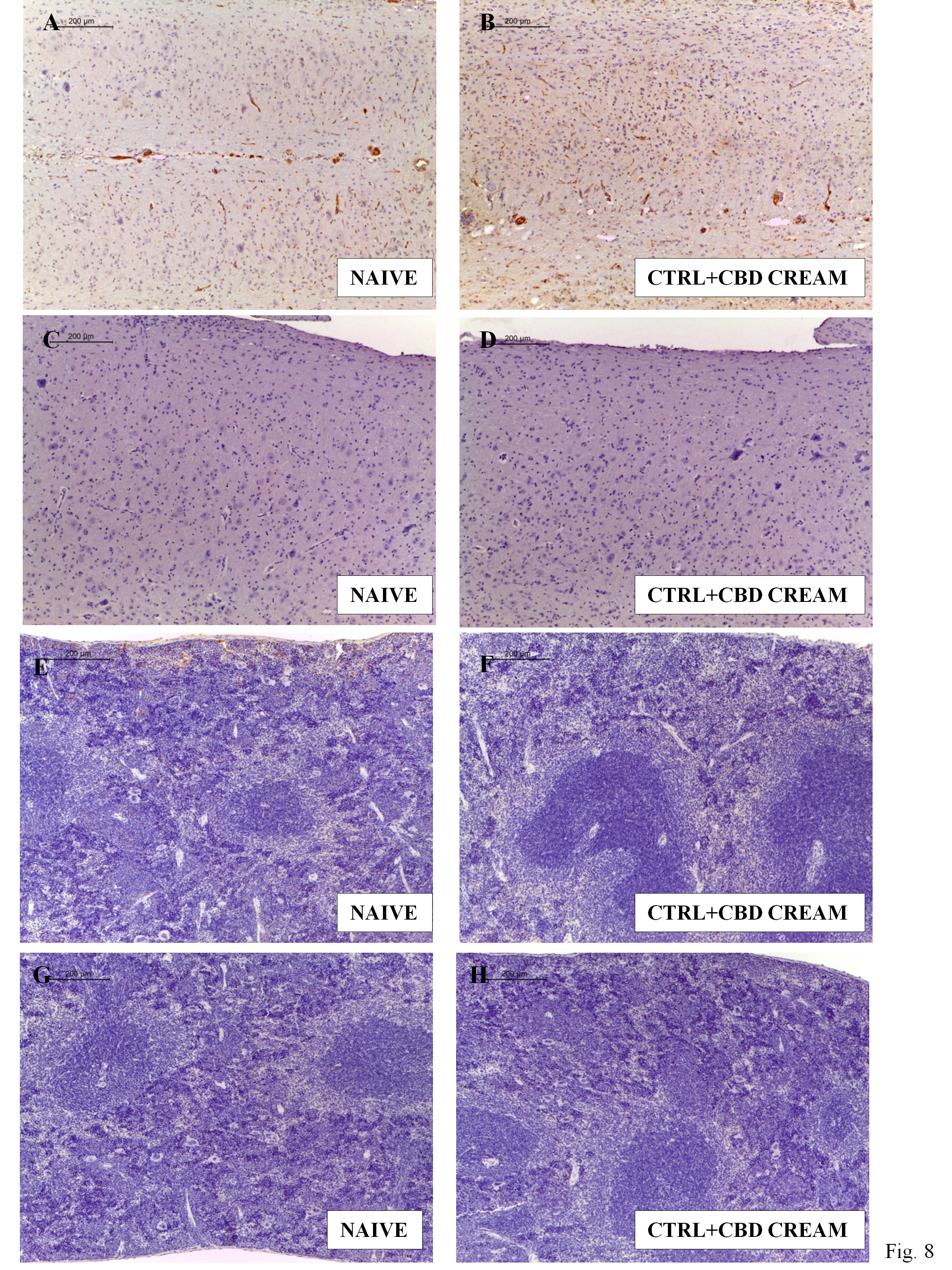

Supplement: Additional file 2: Figure S2. — Immunohistochemical localization for p-selectin in naive mice (A:10x) and CTRL-CBD cream (B:10x). Immunohistochemical analysis for IL-1β in spinal cord tissues from naive mice (C:10x) and for CTRL-CBD cream (D:10x). Immunohistochemical analysis for CD4 in spleen tissues from naive mice (E:10x) and for CTRL-CBD cream (F:10x). Immunohistochemical image for CD8α localization of naive mice (G:10x) and for CTRL-CBD cream (H:10x) in spleen tissues. (TIFF 19661 kb) [file 40199_2015_131_MOESM2_ESM.tif]
